# Minute Structure of an Amalgam-Filled Tooth

**Published:** 1871-06

**Authors:** S. P. Cutler


### SELECTED ARTICLES.

### ARTICLE VIII.

### *Minute Structure of an Amalgam-Filled Tooth.*

By S. P. Cutler, M.D., D.D.S.

This tooth, lower second left bicuspid, decayed on poterior approximal surface, and extending over half of grinding surface, but not quite reaching the nerve, had been filled six months, and had given no trouble until a short time ago, when it became painful and somewhat elongated—pain experienced on occlusion of jaws for the twenty-four hours previous to its removal. The tooth never gave any trouble until a few days before extraction. The young man is about twenty-one years old, of nervous temperament, a clerk in a store. The fang of the tooth, about half way up from apex, presented that peculiar hyaline, or translucent appearance, usually witnessed in old age and in loose teeth, showing, more or less, closing of tubuli, and, more or less, secondary ossification of pulp cavity. It also had a long fang, rather crooked, firm in socket; none of the teeth missing on that side, though many of them were filled with gold and one molar with amalgam. This last at first was supposed to be the offender. The filling was removed, which gave entire relief to that tooth, but soon the pain reappeared with renewed energy in the tooth under consideration. On splitting open the tooth the crown portion of the nerve was found to be in a suppurating condition of recent origin, extending into the neck. The balance was only inflamed.

#### MICROSCOPIC APPEARANCES.

A central longitudinal section gave the following appearances: On the side of pulp cavity, occupied by the plug, secondary dentine had progressed inwards and downwards to a considerable extent as far as the alveolus, becoming gradually narrower, and finally ceasing by gradual taper to the opposite side of normal cavity—the secondary formation passing more than half around the cavity. The dentine over this region, up to decayed surface, translucent, similar to fang, with the usual characteristic change in tubular structure, though not so marked. The balance of crown normal, only the surface of decay which was not entirely removed in excavating, though no signs of decay going on. For a short distance, so far as the discolored *dentine* extends, there is the usual characteristics buccated or beaded appearance in tubuli, not caused by reason of mercury, as that is a common appearance of decayed dentine. I could discover no signs of mercury in tubuli whatever, or any original defects in the dentine. The pulp cavity of fang, to the extent of secondary change, as mentioned above, presented the usual secondary dentification from apex up to about the middle. On all sides about equally narrowing the canal, showing the peculiar irregularities of secondary dentinal tubuli, so often referred to in my former articles. The cement on one side of the fang was hypertrophied up to the middle, or as far as the change in the primary dentine and secondary limits. The usual bone structures of the cement somewhat changed the canalculi, being less distinctly marked. My object in writing this article is to show the effects of filling where the decay is extensive and nerve nearly exposed and sensitive. This tooth had the white form of decay, as I was informed by the operator who filled it. Under the filling, the tooth cavity was dark and firm, the amalgam filling also being dark but sound; dark from oxidation, no doubt. There was a portion of the white softened dentine left intentionally over the nerve as a protection, or rather to avoid exposure of nerve from too much excavating. The oxidizing of the amalgam, no doubt, caused the darkened appearance of the dentine, and arrested any tendency to further decay from therapeutic influence. I examined closely to see if there was any traces of quicksilver in the tubuli or surface of decay or nerve cavity, but failed to find any.

Now, the question is this: is it possible, even where the pulp is dead, for the vapor of quicksilver to enter the minute tubuli by force or *osmose* action, or any other way, as there seems to be no adhesive affinity between the mercury and dentine to allow of what is termed *welting?* This is governed by certain affinities, that is, if the adhesive attraction between a liquid and solid exceeds one-half the cohesive attraction of the liquid, then welting takes place, otherwise not. Now, mercury and gold, silver and tin, possess this property, and if the tooth was composed of similar materials the mercury would readily pass into and through the tubes, if open; but there being no such affinity, no mercury could be found in dentinal tubes. Water will readily enter dentinal tubes, as the requisite affinities there exist. These fine capillary tubes may be supposed to absorb on the same principle as welting. Mercury, glass, porcelain, and some of the metals possess no such affinities. Many other bodies might be also named. Mercury will even dissolve gold, silver, tin and other metals, and when this takes place the *affinity* goes still further than that of welting, and is explained in this way, when any solid body is dissolved in a liquid, or forms a solution, the adhesive affinity between the solid and liquid exceeds the entire affinity of cohesion of the fluid atoms. What are the facts in relation to the tooth under consideration? This tooth had lost a large portion of its crown by decay, which was restored by an amalgam filling. This being a foreign body to the tooth, imparted its shocks through its conducting power, which being abnormal to the tooth caused disturbance to its myriad of nerve fibrils weakening their powers of vital resistance by slow degrees. As the vital resistance ebbs, the mineral encroachments march on apace, that is, as the vital integrity lowers the circulation in the part becomes lessened or stagnated. In consequence, there would be more time allowed for the mineral salts, as lime, to infiltrate into the now enfeebled tissues, and there remain a sufficient time to permit absorption to take place. In this way a nucleus is formed in the pulp substance, which serves, in turn, to attract more atoms around it and continues to grow, similar to the formation around the teeth, forming nodular dentine. But in the above case I found no nodules in the pulp, as I might have expected, on the contrary, I found precisely the same state of things that always takes place in old age, though in a much more hurried manner. In the one case, perhaps, an effect, and in the other, a cause, of lessened vital resistance. This effect, reacting and serving equally as a cause in both cases, bringing about the same results. This, though not yet a settled fact, may answer us as hypothesis, and may ultimately prove a correct theory. In the case cited above, there was of necessity great destruction of nerve fibrils, from the extent of decay lessening the sum total of vital resistance, at least throughout the substantia eburnia, or dentine, allowing the growth inwards in tubes and pulp cavities as already given above. Why the same effect did not take place throughout the entire tooth is not so easily accounted for, as this same partiality is recognizable in all similar cases from whatever cause. Even the changed dentine is not uniform in its character, the portions unchanged always presenting the usual normal appearance, only more yellow in advanced age. In all such cases the tubes are narrowed, more or less, throughout, as well as the entire pulp cavity. At what precise time this process commenced in the above tooth is not easy to determine. It may or may not have commenced before filling, though from the numerous observations I have made in teeth similarly decayed, I am led to believe the change took place after filling. How much depended on the influence of the mercury I cannot say, neither can I say that gold would have done any better, or even tin; each might be governed by relative conducting powers. Whether the battery influence of the amalgam had any agency in the process, must be decided by more extended observation. It is possible that some non-conducting substance, such as gutta percha, or Hill's stopping, used as a filling, or as a capping under a metallic filling, would offer greater protection to the beleagured pulp beneath. O-sartificial is a non-conductor, but on first application is irritating, from the action of the hydrochloric acid on the lime of the tooth. The greater affinity of the acid for the lime, than for the zinc, decomposes a superficial layer, and in consequence renders that layer chalky. This becomes manifest through the enamel when in contact with it. This I propose to remedy by first introducing styptic colloid into the cavity on cotton and letting it remain a few moments, then remove the cotton, and by the time the os-artificial can be introduced into the tooth the ether is evaporated, leaving an impervious coating which the acid has no power to act on, in this way permanently projecting the lime of the tooth. Non-conductors also have their objections, as in the case of rubber plates. In gaining one point we may lose another, so that we have much to learn yet on the subjects of fillings in large cavities. Os-artificial, like every new lion in the arena, needs taming before it can be regarded as perfectly harmless under all circumstances.

In the above case there might have still been a chance for healthy action in the process of secondary dentification, could the process have been conducted a little slower, so as to have allowed sufficient time for pulp absorption, so that it could have been removed out of the way in time, and not have been too much crowded in among delicate nerve fibrils, causing inflammation and its consequences as above named.

The other day I extracted a molar for a young man which was decayed nearly to the nerve and aching. On splitting it open I found the bulb, or pulp, nodular, and no signs of inflammation. The pulp had a white appearance, and was unusually dense and plump throughout. Where the nodules were not, it was evidently thoroughly saturated with lime salts, so much so as to prevent any undue distinction of vessels and consequent inflammation. This tooth ached from pressure of earthy matter among the nerve fibrils, though not of an acute character, as in cases of actual inflammation of pulp. In a short time the entire pulp must have become ossified and ultimately subsided into quietude. The investing pericementum was inflamed, which was another source of trouble. All such teeth possibly could be saved by proper treatment, by assisting nature a little in her difficulties. This is only one, out of a great many that I have examined that were in a similar condition, that I might mention. I am not aware that other writers have paid much attention to this condition of pulp but only the nodular condition. This saturation of lime salts I have been studying carefully for some time, with a view to restoring such teeth, when troublesome, to a healthy condition. This is in many cases practicable, where the patient is manageable, or willing to wait and endure for a time a little inconvenience. There may be skeptics on this question, as well as on other unsettled points, but they are generally instigators and open to conviction. My ideas concerning the cause of this condition of pulp have been repeatedly given to the profession, but I will recapitulate a little. In the first place, there must be a weakening of vital resistance in the part before any such encroachments can occur. Then a stasis in the circulation takes place from want of capillary integrity of function; or, in other words, *defective pronouncement* on their part, owing to neural or vital weakness, as the capillaries have a special function to to perform. After stagnation in the vessels for some time, the lime salts begin to osmose into the extra vascular tissue, and thereby, desiccating or drying, they begin to thinken and ultimately harden into nodules, sometimes filling the entire pulp, as in the case cited above, and by their pressure forcing the blood-vessels to deplete themselves, and by pressure pain is produced.

In the treatment of such cases, in the first instance, we must restore vitality to the pulp, which may be done by local stimulants of various kinds, including galvanism. This may be readily applied. At the same time weak vegetable acids might be used on cotton sealed up in the cavity. The acid should not be strong enough to decompose the dentine. This may be alternated with carbolic acid, creasote and various other agents. The object of the acid treatment is to dissolve out the soft solid lime salts from pulp : vinegar will do for this purpose. These ideas are suggestive only, not being based on very extensive experience with such teeth in the mouth, but outside with favorable results. I have treated many teeth successfully where I believed this acidifying process was going on; I have one of the kind in my own mouth, which gave me trouble, though not decayed. We often see cases in young subjects, where the teeth are very much crowded and lapped, causing trouble from pressure one on the other sufficiently to cause nodulation.

We often cap and fill teeth where there is slight exposure, sometimes with success and at others with failure and subsequent death of pulp. Most of the successful cases, sooner or later, undergo ossification of cavity or pulp, or both, to a greater or less extent. Sometimes such cases, from hurried action, are lost, when they might be saved by filling.

The lime salts, previously mentioned, after saturating the pulp, more or less, from pressure, drives the blood slowly and gradually out of the tooth, the salts taking the place of the blood in the vessels as the blood leaves them, and the entire pulp becomes differentiated into secondary dentine. The nerve fibrils alone resist this process, and in some cases, succomb under the extent of earthy influence and suffer more or less ossification, the pressure causing their absorption.

One very remarkable and noticeable feature in the pulp of an aching tooth, where it is saturated with the earthy salts, is a total want of all signs of inflammation in such pulp, even where only a portion is saturated, which has a chalky feel and appearance. This chalky appearance always precedes nodulation. When this is perfected, the noduli becoms translucent, and lose the white appearance. In a few exceptionable cases the pulp remains perfectly white.—*Dental Times.*

